# Tunneling Nanotubes and Gap Junctions–Their Role in Long-Range Intercellular Communication during Development, Health, and Disease Conditions

**DOI:** 10.3389/fnmol.2017.00333

**Published:** 2017-10-17

**Authors:** Jennifer Ariazi, Andrew Benowitz, Vern De Biasi, Monique L. Den Boer, Stephanie Cherqui, Haifeng Cui, Nathalie Douillet, Eliseo A. Eugenin, David Favre, Spencer Goodman, Karine Gousset, Dorit Hanein, David I. Israel, Shunsuke Kimura, Robert B. Kirkpatrick, Nastaran Kuhn, Claire Jeong, Emil Lou, Robbie Mailliard, Stephen Maio, George Okafo, Matthias Osswald, Jennifer Pasquier, Roel Polak, Gabriele Pradel, Bob de Rooij, Peter Schaeffer, Vytenis A. Skeberdis, Ian F. Smith, Ahmad Tanveer, Niels Volkmann, Zhenhua Wu, Chiara Zurzolo

**Affiliations:** ^1^GlaxoSmithKline, Collegeville, PA, United States; ^2^Department of Pediatric Oncology, Erasmus MC – Sophia Children's Hospital, Rotterdam, Netherlands; ^3^Division of Genetics, Department of Pediatrics, University of California, San Diego, La Jolla, CA, United States; ^4^GlaxoSmithKline, Stevenage, United Kingdom; ^5^Public Health Research Institute (PHRI), Newark, NJ, United States; ^6^Department of Microbiology, Biochemistry and Molecular Genetics, Rutgers New Jersey Medical School, Rutgers the State University of New Jersey, Newark, NJ, United States; ^7^GlaxoSmithKline, Research Triangle Park, NC, United States; ^8^Department of Biology, College of Science and Math, California State University, Fresno, CA, United States; ^9^Bioinformatics and System Biology Program, Sanford Burnham Prebys Medical Discovery, La Jolla, CA, United States; ^10^GlaxoSmithKline, Waltham, MA, United States; ^11^Laboratory of Histology and Cytology, Graduate School of Medicine, Hokkaido University, Sapporo, Japan; ^12^Division of Cancer Biology, Physical Sciences-Oncology Network, Cancer Tissue Engineering Collaborative Research Program, Program Director, Structural Biology and Molecular Applications Branch, National Cancer Institute, Bethesda, MD, United States; ^13^GlaxoSmithKline, King of Prussia, PA, United States; ^14^Division of Hematology, Oncology and Transplantation, Department of Medicine, University of Minnesota, Minneapolis, MN, United States; ^15^Department of Infectious Diseases and Microbiology, University of Pittsburgh, Pittsburgh, PA, United States; ^16^Neurology Clinic and National Center for Tumor Diseases, University Hospital Heidelberg, Heidelberg, Germany; ^17^Clinical Cooperation Unit Neurooncology, German Cancer Consortium (DKTK), German Cancer Research Center (DKFZ), Heidelberg, Germany; ^18^Department of Genetic Medicine, Weill Cornell Medical College in Qatar, Qatar Foundation, Ar-Rayyan, Qatar; ^19^Division of Cellular and Applied Infection Biology, RWTH Aachen University, Aachen, Germany; ^20^Institute of Cardiology, Lithuanian University of Health Sciences, Kaunas, Lithuania; ^21^Department of Neurobiology and Behavior, University of California, Irvine, Irvine, CA, United States; ^22^Section of Intracellular Trafficking and Neurovirology, National Institute of Health, Bethesda, MD, United States; ^23^Unit of Membrane Trafficking and Pathogenesis, Department of Cell Biology and Infection, Pasteur Institute, Paris, France

**Keywords:** Alzheimer, inflammation, cancer, gap junctions, reactivation

## Abstract

Cell-to-cell communication is essential for the organization, coordination, and development of cellular networks and multi-cellular systems. Intercellular communication is mediated by soluble factors (including growth factors, neurotransmitters, and cytokines/chemokines), gap junctions, exosomes and recently described tunneling nanotubes (TNTs). It is unknown whether a combination of these communication mechanisms such as TNTs and gap junctions may be important, but further research is required. TNTs are long cytoplasmic bridges that enable long-range, directed communication between connected cells. The proposed functions of TNTs are diverse and not well understood but have been shown to include the cell-to-cell transfer of vesicles, organelles, electrical stimuli and small molecules. However, the exact role of TNTs and gap junctions for intercellular communication and their impact on disease is still uncertain and thus, the subject of much debate. The combined data from numerous laboratories indicate that some TNT mediate a long-range gap junctional communication to coordinate metabolism and signaling, in relation to infectious, genetic, metabolic, cancer, and age-related diseases. This review aims to describe the current knowledge, challenges and future perspectives to characterize and explore this new intercellular communication system and to design TNT-based therapeutic strategies.

## Introduction

Cell-to-cell communication is essential to all biological processes. Tunneling nanotubes (TNTs), also named cytonemes and tumor microtubes, are a recently discovered form of the long-distance communication system between cells (Onfelt et al., 2004; Rustom et al., 2004; Gerdes et al., 2007). Consisting of long cytoplasmic, open-ended or connexin-containing protrusions that can connect cells, the proposed functions of these structures are diverse and have been shown to include the long-range exchange of organelles, vesicles, and small molecules between connected cells (Gerdes et al., 2007). Data from *in vitro* and *ex vivo* studies indicate that TNTs are minimally observed in uninfected cells (Eugenin et al., 2009a; Gerdes et al., 2013). In contrast, *in vitro* TNT formation and TNT-mediated intercellular communication are significantly higher in several pathologic forms of disease, including, virus infection, cancer, synucleinopathies (Parkinson's disease, Lewy bodies, and multiple system atrophy) as well as tauopathies, and prion-associated diseases (Gerdes and Carvalho, 2008; Eugenin et al., 2009a; Gousset et al., 2009; Abounit and Zurzolo, 2012; Wang and Gerdes, 2012; Gerdes et al., 2013; Austefjord et al., 2014; Abounit et al., 2015, 2016a,b; Desir et al., 2016; Tardivel et al., 2016). Several laboratories observed the presence of connexin and gap junction channels in TNTs, but the role of gap junctions (GJ) in these processes and these diseases is still under active investigation. These observations open the possibility of a long-range gap junctional communication mediated by the TNT processes.

In pathological conditions, TNT numbers can increase and facilitate the intercellular spread of infectious and toxic agents. To date, TNT formation has been observed in tissue culture in many different mammalian cell types (from epithelial to endothelial, mesenchymal and stem cells), immune cells (including B, T, NK cells, neutrophils, monocyte/macrophages and dendritic cells), neurons, glial cells and cancer cells, suggesting that their presence is more ubiquitous than initially thought (see review by Gerdes et al., 2007). *In vivo*, TNT-like protrusions called cytonemes have been observed in the imaginal disc development of *Drosophila* (Kornberg, 1999; Hsiung et al., 2005) and prior to fertilization of *Plasmodium* gametes in the midgut of the *Anopheles* malaria vector (Rupp et al., 2011). Malaria parasites form filamentous cell-to-cell connections during reproduction in the mosquito midgut (Rupp et al., 2011). Furthermore, TNT-like structures have been commonly observed between immune cells in lymph nodes (see review by Onfelt et al., 2004; Gerdes et al., 2007; Zaccard et al., 2016) and between dendritic cells in mouse cornea (Chinnery et al., 2008). Other examples of TNT-like structures observed in tissues have been reported in malignant tumors resected from human cancer patients (Pasquier et al., 2013; Ady et al., 2014; Antanaviciute et al., 2014; Thayanithy et al., 2014b), in leukemic cells obtained from bone marrow aspirates of pediatric patients (Polak et al., 2015) and in cardiac myocytes and non-myocyte cells in heart damage (Quinn et al., 2016). Moreover, an impressive *in vivo* demonstration of TNT-like structures (named tumor microtubes, TMs) has been reported in malignant gliomas, providing further support for a potentially important role for direct intercellular communication by TNT and GJ in tumor development and progression (Osswald et al., 2016). Interestingly, Dr. Gerdes's laboratory demonstrate that TNT between different cell types are electrically coupled by a mechanism involving gap junctions (Wang et al., 2010, 2012; Wang and Gerdes, 2012; Gerdes et al., 2013; Austefjord et al., 2014).

On September 22-23, 2016, academic leaders in the TNT field (see authors list) met in Collegeville, Pennsylvania, USA to discuss “*Tunneling nanotubes (TNTs): Cell to Cell Social Networking in Disease*.” In addition to the basic biology experts from Europe, Asia, and the United States, the meeting had extensive interest and attendance from researchers from the pharmaceutical industry, and the U.S. National Institutes of Health (NIH); this unique combination of basic and translational research expertise produced vigorous discussion and debate on several important aspects of this new field of the biology of intercellular communication including TNT and the role of GJ in health and disease. The focus was to clarify what defines TNT structures, what signals trigger their formation and accountability for their differential permeability and selectivity. Lastly, the potential use of TNTs to rescue cells from cell death or metabolic distress and as novel therapeutic approaches were considered. The conclusions drawn from the discussions are summarized in this review.

### TNT identity

In the last 10 years, there have been many descriptions and observations of cellular protrusions connecting cells, which appear quite different from TNTs. Hence, it is critical to be able to distinguish TNTs from other types of cell projections. The similarity between TNT and GJ channels were highlighted (Rustom et al., 2004; Watkins and Salter, 2005). Some TNTs have been shown to possess GJ components (Wang et al., 2010; Wang and Gerdes, 2012). Data from Drs. Osswald and Eugenin showed that connexin-43 (Cx43) is present in the TNT-like structures under various contexts (between astrocytoma cells or between macrophages) and that inhibition of GJ channels does not prevent their formation but does interfere with normal communication between TNT connected cells. These data suggest that the two communication systems evolved to complement each other in coordinating cell-to-cell communication.

A related issue is whether all TNTs are open-ended and what is the mechanism of their formation. Some reports described the intercellular exchange of Ca^2+^ signals between distant cells are mediated via TNTs (Watkins and Salter, 2005; Hase et al., 2009; Wang et al., 2010, 2012; He et al., 2011; Smith et al., 2011; Wittig et al., 2012; Al Heialy et al., 2015; Osswald et al., 2015) suggesting some form of membrane/cytosolic continuity along these structures or active GJ channels are present at the end of the process (Wang et al., 2010, 2012; Wang and Gerdes, 2012; Gerdes et al., 2013; Austefjord et al., 2014). The mechanisms involved in this process of intercellular Ca^2+^ wave propagation are not well understood, but GJ are thought to be intimately involved (Wang et al., 2010, 2012; Wittig et al., 2012; Lock et al., 2016). Further, the observation in lymphocytes that TNTs are not permeable to Ca^2+^ highlight the diverse phenotype in their physiological properties (Davis and Sowinski, 2008; Sowinski et al., 2008). Characterization of TNTs in untreated cells in culture indicates that TNTs are uniformly F-actin positive and have low expression of tubulin (Onfelt et al., 2006; Rupp et al., 2011; Gousset et al., 2013; Thayanithy et al., 2014a; Astanina et al., 2015; Polak et al., 2015) suggesting that actin regulators and actin-driven motors might be implicated in the formation and/or function of TNTs. In PC12 cells, the immunocytochemical analysis demonstrates that synaptophysin, a marker of synaptic vesicles, as well as Myosin-X (Myo10) and Va (MyoVa), both actin-based motor proteins, were present inside TNTs (Rustom et al., 2004). These data were confirmed in other cell types (Gousset et al., 2013; Schiller et al., 2013; Reichert et al., 2016; Tardivel et al., 2016), and M-Sec through Ral-mediated actin remodeling was shown to be involved in TNT formation as reported by Dr. Kimura (Hase et al., 2009; Ohno et al., 2010). Furthermore, recent data indicated that TNT mediates a long-range transmission of IP_3_ by a gap junction-dependent mechanism (Lock et al., 2016). Nonetheless, it is still entirely unknown which proteins are involved in the formation, stability, and transport associated with TNTs and is very likely that different mechanisms will participate in the formation of these structures and are prevalent in different cell types.

Filamentous Actin (F-Actin), M-Sec, myosin Va, and X, as well as Cx43, are well-known components of TNTs, and the blocking any of these components reduces or prevents communication. Preliminary data from Dr. Den Boer showed that various types of actin inhibitors, but not tubulin inhibitors, will reduce the level of TNT signaling in leukemia. Novel data from Dr. Zurzolo showed that TNTs and filopodial extensions (which look very similar in confocal microscopy) have different requirements and rely on different actin regulators (Abounit et al., 2015). This is consistent with the previous observation made from the same group (Gousset et al., 2013).

Several groups have demonstrated that HIV-infected cells (e.g., those containing proteins or infected with HIV) can send TNTs to neighboring uninfected or healthy cells, resulting in the spread of infection or aggregation of toxic viral proteins. Dr. Gousset indicated that the transfer of the HIV-1 Nef accessory protein is mediated *via* TNTs between a macrophage cell line and T cells. Using this Nef model system, it was shown that Nef transfer occurred through a Myo10-dependent mechanism. Similarly, diseased cells lacking functional lysosomes have also been shown to induce TNT formation from nearby healthy cells to facilitate lysosome delivery into diseases cells (Abounit et al., 2015, 2016a). Interestingly, lysosomal dysfunction occurs in neurodegenerative disease. Dr. Zurzolo's group recently showed that lysosomes could be transferred through TNTs to mediate the intercellular spreading of misfolded alpha-synuclein in a neuronal cell model of Parkinson's disease (Abounit et al., 2015, 2016b). Lysosomal cross-correction *via* TNTs was also shown in the context of a lysosomal storage disorder after hematopoietic stem cell transplantation resulting in long-term tissue preservation (Yasuda et al., 2011; Astanina et al., 2015; Naphade et al., 2015; Abounit et al., 2016a). Similar TNT transfer mechanisms have been observed for mitochondria in different diseases (Han et al., 2016; Jackson et al., 2016; Jiang et al., 2016; Reichert et al., 2016; Sinclair et al., 2016; Wang et al., 2016; Zhang et al., 2016).

**Figure 1:**
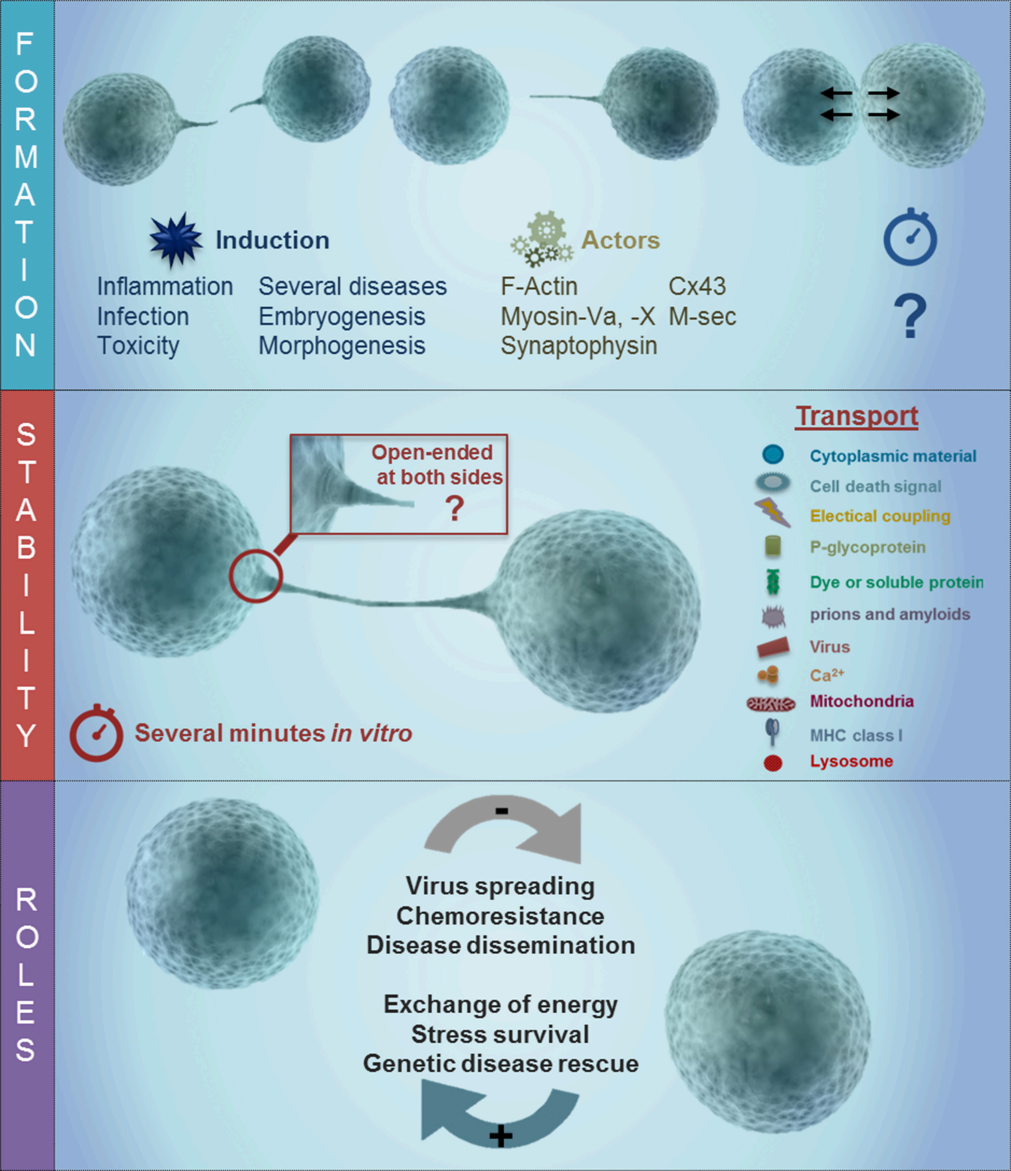
Schematic of TNT formation and the potential role of gap junction channels during long rage communication. As described in the text, TNT have at least 3 different stages, including formation, stabilization, and the transport of the cargo. The last one is associated with several different roles in disease including viral spreading, chemoresistance, and disease dissemination as well as an energy associated survival, genetic disease rescue and stress survival. TNT formation is triggered by inflammation, infection, toxicity, in several disease, and embryogenesis/morphogenesis. Some of the proteins involved in the formation of TNT are actin, Myosin Va and X, synaptophysin, Cx43, and M-sec. Following the formation of the TNT process, there are at least 2 different types of tubes, a synaptic and open-ended process. The formation of these long rage TNT enable the connected cells to share multiple proteins and lipids.

The intracellular and extracellular signals involved in the formation, permeability, and directionality of these TNTs are unknown. Interestingly, experiments using different tumor cell lines, primary astrocytes, acute leukemia cells, T cells, and macrophages demonstrate that the formation, communication, transfer of metabolites and the collapse of the TNTs are extremely fast (30–60 s) and can reach distances up to 300 μm. To further understand the properties of TNTs and GJ either the identification of novel proteins and lipids capable of supporting these mechanisms or identification of new TNT-related functions of existing proteins are required. The main conclusion was that several types of TNTs are present in multiple cell types and tissues. Further research is required to identify potential biomarkers of TNT formation for different cell types is therefore warranted. Moreover, an agreed definition of a TNT has been the subject of much debate and consensus amongst TNT scientists is a tubular membrane connection between non-adjacent cells that allow direct intercellular communication, not necessarily gap junction-mediated. They contain F-actin, are open-ended and have a variable diameter from 50 to 800 nm. Although different types of tubular, membranous connections have been observed to form between distant cells, the term “TNT-like structure” can be ascribed to these cellular structures, provided that they fulfill the essential requirement of allowing intercellular exchanges of any material, (e.g., vesicular, particulate, ionic, molecular, organismic) between the connected cells (see Figure 1). To identify TNT-associated structures, there is a need for new or improved super-resolution and electron microscopy methods that can structurally characterize this new intercellular communication system in more detail. It will also be important to describe TNTs in different cell types and situations, where expression of one TNT type may predominate. Also, more data using live imaging systems are needed to describe the mechanism of transfer.

### TNTs in the healthy vs. diseased state

Another important question under consideration is the timing and location of TNT formation. Several reports indicated that viruses, such as herpes (La Boissiere et al., 2004; Sherer et al., 2007), influenza (Kumar et al., 2017), and pseudorabies viruses (Favoreel et al., 2005), can be transmitted through long extensions without contact with the extracellular environment, suggesting that viruses may have evolved to use TNTs to spread efficiently between connected cells (Figure 1). The signals that guide the formation of TNTs are not entirely known. However, a re-examination of older reports through the prism of the current knowledge of TNTs indicates that there were published descriptions of increased formation of TNT-like structures in inflammatory conditions. In particular, TNT-like structures have been observed under the following pathological conditions *in vitro*: cell infected with *Listeria monocytogenes* and *Mycobacterium Bovis* (Dramsi and Cossart, 1998; Wehland and Carl, 1998; Onfelt et al., 2006), in astrocytes treated with H_2_O_2_ (Zhu et al., 2005), microglia activated with PMA and calcium ionophore (Martinez et al., 2002), monocyte/macrophages treated with LPS plus IFN-γ (Eugenin et al., 2003), lymphocytes and human macrophages infected with HIV (Sowinski et al., 2008; Eugenin et al., 2009b), mouse neuronal CAD cells and primary neurons and astrocytes infected with exogenous PrP (Gousset et al., 2009, 2013), and more recently neurons treated with pathogenic amyloid aggregates (Costanzo et al., 2013; Abounit et al., 2016a,b). It is therefore not surprising that TNT-like structures have also been identified in normal hematopoietic (CD34^+^) progenitor cells and lymphoid leukemia cells and that interference with TNT signaling in the hematopoietic context results in altered secretion of cytokines (Polak et al., 2015). Interestingly, most of these treatments are also associated with the formation and functional gap junctional communication, especially in immune cells.

Dr. Zurzolo's group proposed that diseases associated with the spread of the misfolded aggregated proteins within the CNS (like a prion, Alzheimer, Parkinson, and Huntington disease) might involve TNT-mediated spreading (Abounit and Zurzolo, 2012; Delage and Zurzolo, 2013; Abounit et al., 2015, 2016a,b; Delage et al., 2016). They demonstrated that prion protein, PolyQ Huntingtin, fibrillar tau and alpha-synuclein transfer between neurons in culture using TNTs as the predominant mechanism of dissemination (Abounit et al., 2016a,b). Together with the postulated role of TNTs in HIV spreading within the central nervous system (Eugenin et al., 2009a,b; Abounit et al., 2016a), this suggests that multiple diseases can use TNTs to spread toxicity and infection, identifying TNTs as an exciting new potential therapeutic target. Indeed, inhibition of TNTs may block or reduce the amplification of several diseases including HIV, Parkinson's disease, Lewy bodies, and multiple system atrophy as well as tauopathies (Gousset et al., 2009, 2013; Abounit and Zurzolo, 2012; Costanzo et al., 2013; Abounit et al., 2016a,b). Dr. Zurzolo presented data showing that misfolded aggregated tau leads to an increase in TNT formation in culture, but the role of gap junction channels in these tubes was not examined (Abounit et al., 2016b). In agreement with Dr. Zurzolo's findings, several groups have identified TNT like structures in tau related pathologies and their potential role in disease by facilitating electrical coupling and calcium signaling between distant cells (Gerdes et al., 2007; Wang et al., 2012; Wittig et al., 2012; Tardivel et al., 2016), supporting further a potential role of gap junction channels in TNT biology.

Another important role of TNTs in disease may be linked to modulation of the tumor microenvironment. Data from Dr. Den Boer showed that TNTs are actively formed between leukemic cells and bone marrow-derived mesenchymal stromal cells. This interaction is beneficial to the viability of leukemic cells and induces chemo-resistance, which can be abrogated by disrupting the TNTs (Polak et al., 2015). Only recently, Drs. Winkler and Osswald demonstrated that TNT-like structures are essential in the pathogenesis of astrocytomas including the participation of connexin containing channels (Osswald et al., 2015, 2016; Winkler, 2016; Jung et al., 2017; Weil et al., 2017).

As indicated above, the exact role of TNTs and GJ channels is unclear. However, there is evidence that a specific type of TNT-like structures (called cytonemes) have been observed during developmental stages of several organisms like *Drosophila* and have been postulated to play a role in embryonic development, differentiation, and morphogenesis (Ramirez-Weber and Kornberg, 1999; Roy et al., 2011, 2014; Rojas-Rios et al., 2012; Bilioni et al., 2013; Bischoff et al., 2013; Kornberg, 2014; Kornberg and Roy, 2014; Huang and Kornberg, 2016; Karlikow et al., 2016). Further, TNT-like structures were found in the unicellular malaria parasites during gametogenesis, which takes place in the midgut of the *Anopheles* mosquito and proposed to be important for the initial contact between mating partners (Rupp et al., 2011). Although the role of TNTs in normal cells was not specifically addressed, there is a large body of data supporting the presence and the need of TNT-like communication during development and immune cell activation (Kornberg, 1999; Ramirez-Weber and Kornberg, 1999; Roy et al., 2011, 2014; Bilioni et al., 2013; Bischoff et al., 2013; Briscoe and Vincent, 2013; Polak et al., 2015; Huang and Kornberg, 2016; Karlikow et al., 2016). A recent report from the Mailliard group describes the induction and regulation of TNTs in dendritic cells as a normal component of their function as mediators of adaptive immunity (Zaccard et al., 2015). In this study, dendritic cells matured under type-1 pro-inflammatory conditions acquired a unique program to rapidly form intercellular networks of tunneling nanotube-like structures upon subsequent antigen-driven interaction with CD4^+^ T-helper (T_H_) cells. This immune process, which they termed dendritic cell “reticulation,” is induced by the T_H_ cell-derived factor CD40L, and serves to facilitate the functional intercellular transfer of antigens and endosomal vesicles (Zaccard et al., 2015). Interestingly, this process is differentially regulated by the opposing activity of the respective T_H1_- and T_H2_-associated cytokines IFN-γ and IL-4. Importantly, they also describe how the induction and regulation of TNT networks in dendritic cells can be exploited by pathogens such as HIV to facilitate cell-to-cell spread (Mailliard et al., 2013; Zaccard et al., 2015). Similar results of antigen sharing has been described in the context of GJ communication (Neijssen et al., 2005; Matsue et al., 2006; Corvalan et al., 2007; Handel et al., 2007; Mendoza-Naranjo et al., 2007; Pang et al., 2009). Thus, it may be that a similar mechanism of amplification of the immune response can be mediated either by TNT's or by gap junctions.

Under inflammatory or pathological conditions in the context of a genetic lysosomal storage disorder, cystinosis, TNTs also serve as a delivery system to transfer “healthy” lysosomes. Indeed, following the systemic transplantation of wild-type hematopoietic stem and progenitor cells (HSPCs) in the mouse model of cystinosis, Ctns^−/−^ mice, HSPCs differentiate into macrophages and generate TNTs that transfer cystinosis-bearing lysosomes to the adjacent disease cells, leading to long-term kidney preservation (Naphade et al., 2015). A similar mechanism accounts for the conservation of the cornea and thyroid in the Ctns^−/−^ mice (Rocca et al., 2015; Gaide Chevronnay et al., 2016). An understanding of the role of this new communication system in quiescent cells, during the immune response and in pathological conditions may open new potential therapeutic opportunities to target these diseases with none-to-minimal side effects as the current scientific data suggests that TNTs are only minimally expressed under homeostatic conditions.

### TNT's in transport

Another important question in the TNT field concerns the types of cargos being transported within the TNTs. Several reports support the idea that different types of TNTs, as categorized by size, content, and permeability, exist in different cells and under different conditions as well as presence or absence of gap junction channels. TNTs have been shown to mediate long-range transmission of Ca^2+^ signals between cells (Watkins and Salter, 2005; Hase et al., 2009; Wang et al., 2010, 2012; He et al., 2011; Smith et al., 2011; Wittig et al., 2012; Al Heialy et al., 2015; Osswald et al., 2015), a novel mechanism that adds to the known repertoire by which Ca^2+^ ions communicate information between cells. The mechanism through which this occurs is not well understood, but gap junctions are thought to play a role in mediating intercellular transmission of Ca^2+^ waves (Wang et al., 2010, 2012; Wittig et al., 2012; Lock et al., 2016). In other instances, TNT has been shown not to be permeable to Ca^2+^. TNTs in other systems allow transport of mitochondria and vesicles, suggesting that the internal pore size is large enough for the trafficking of these organelles (see review by Gerdes et al., 2007; Sherer et al., 2007). The observation that mitochondria can be exchanged between TNT-connected cells is extremely important because it could be one of the first demonstrations of cell to cell transfer of genetic material between non-dividing mammalian cells, suggesting that at least mitochondrial DNA (and potentially siRNA) is not cell type specific and can be shared between different types of cells connected by TNT-like structures (Li et al., 2014; Jackson et al., 2016; Jiang et al., 2016; Sinclair et al., 2016). It is still unclear whether multiple types of TNTs exist or whether the observed differences represent different maturation stages of the same processes. Also, the timing of gap junction formation in relation to the formation of TNT's is no known.

There are two hypotheses that describe how pathogens are sorted in TNTs in infected cells: First, that type of TNT determines the function of the tubular process and type of cargo transported and second, whether TNTs have the capability to sort the cargo at the initiating and terminating regions of the TNT. Both possibilities are feasible based on several scientific papers demonstrating differential TNT selectivity and transport properties (see Figure 1). For example, Drs. Osswald and Eugenin showed that gap junction channels are present in TNTs/TNT-like structures, suggesting that at least this type of TNT may have a cutoff of 1.2 kDa, such that only small molecules can be transferred between TNT connected cells expressing this kind of channel. Dr. Zurzolo showed that PrP^Sc^ (the pathogenic form of the prion protein) and other protein aggregates, as well as organelles and lysosomes, can be transmitted between the connected cells. Recent data from Drs. Lou, Pasquier, Osswald and Den Boer demonstrated that TNTs or TNT-like structures might also play a critical role in tumor growth, metastasis, and chemo-resistance, suggesting that TNT communication in tumors can exchange molecules which accelerate the spreading of disease and induce therapy resistance.

In conclusion, TNT's can transport a variety of products from second messengers (e.g., mRNA to large organelles), but the mechanism of selectivity, transport, and delivery are still unknown. Although myosin motors have been found inside TNTs and therefore likely to be involved in the movement of the different cargoes on the actin cables running inside TNTs, there are still many open questions relating to the identities of the specific motors; whether there is diffusion allowed, and regulation/determination of the different uni- or bi-directional transport mechanisms at play. To answer these questions, fundamental research is required (and should be actively encouraged) to better understand the biology of the structure and composition of TNTs and associated GJ channels, and their potential role in human disease.

### TNT existence *in vivo*

Evidence of TNTs *in vivo* is the central requirement to further progression of research in this area. Literature evidence for the existence of these cellular protrusions has been limited to date, mainly because there are no known specific biomarkers of TNTs. However, a review of the literature revealed several examples of TNT-like structures that have been observed *in vivo* or *ex vivo*. These include the cytonemes found in *Drosophila* (Kornberg, 1999; Hsiung et al., 2005). TNT like structures between immune cells in lymph nodes (see review by Onfelt et al., 2004; Gerdes et al., 2007), and between MHC class II^+^ cells in the mouse cornea (Chinnery et al., 2008), as well as the bridges TNT-like structures observed in several models of malignant tumors (cancer) such as mesothelioma, lung cancer, ovarian cancer, and laryngeal cancer (Ady et al., 2014; Antanaviciute et al., 2014, 2015; Thayanithy et al., 2014b; Desir et al., 2016) or capable of crossing the dense tubular basement membrane in the kidney of the cystinosis mouse model (Naphade et al., 2015) or in their cornea and thyroid (Rocca et al., 2015; Gaide Chevronnay et al., 2016). One major issue in performing these *in vivo* and *ex vivo* studies is the difficulties in identifying the precise nature of the structures and clearly determining their role in the transfer. Nonetheless, several reports have provided *in vivo* evidence to support the role of TNTs in pathophysiology and several forms of the disease. Data from Drs. Osswald, Goodman, Lou, Eugenin and Den Boer reported evidence of TNT-like structures in brain tumors, and in *ex vivo* hematopoietic stem cells, lung, and ovarian cancers. Also, TNT-like structures were found in human macrophages present in lymph nodes obtained from HIV-infected individuals with HIV reactivation.

Interestingly, viruses, such as African Swine Fever, Ebola, Herpes Simplex, Marburg filoviruses, and Poxvirus Vaccinia, encode viral factors or alter cell activation to induce the formation of filopodia structures that allow viral trafficking between the extracellular matrix and environment into cells (Cudmore et al., 1995; Favoreel et al., 2005; Hartlieb and Weissenhorn, 2006; Jouvenet et al., 2006; Kolesnikova et al., 2007; Gill et al., 2008). These observations suggest that viruses have adapted to use TNT-like structures and GJ to promote viral spread. In conclusion, for TNTs to be considered a viable and functional mechanism for intercellular communications, generating compelling *in vivo* data that demonstrate a clear difference between healthy and disease states is critically important.

### TNT and therapy

TNTs are considered to have two potential roles, as a mechanism for spreading disease-forming cargos (from prion to viruses) and/or as a means of spread chemotherapeutic agents, beneficial organelles or cellular molecules during stress and pathological conditions. In diseased cells, TNT levels are significantly elevated which may make it possible to specifically block TNT-like related pathways that are induced only in disease. Data from Drs. Lou, Pasquier and Den Boer proposed several models by which TNT formation and function between cancerous cells may be altered or modulated following response to chemotherapeutic drugs, the following exposure to clinically relevant tumor conditions such as hypoxia, micro-environmental-induced changes and/or following intercellular transfer of cellular organelles, such as mitochondria, microRNAs, and endosomal vesicles or even exosomes. Moreover, under normal conditions, disease states that promote inflammation (especially in cancer) could induce TNT formation in response to metabolic stress (Rustom et al., 2004; Abounit et al., 2016a,b).

TNT formation and induction has also been observed following injury, trauma or chronic tissue stresses. Here, they are thought to play a role in the exchange of energetic components and mitochondria (Wang et al., 2011; Zhang, 2011; Pasquier et al., 2013; Las and Shirihai, 2014; Li et al., 2014; Thayanithy et al., 2014b) to help compromised cells to survive stress. This possibility opens new potential therapeutic opportunities. For example, during the stroke, ischemia and reperfusion conditions regulating the formation of TNTs may provide a means of cell rescue. Furthermore, TNTs offer a novel delivery route for stem-cell based therapies against genetic conditions resulting in organelle dysfunction (Bruzauskaite et al., 2016; Antanavičiūtė et al., 2017) and for chemotherapeutic drugs that disrupt DNA replication, such as nucleoside analogs (Bruzauskaite et al., 2016; Antanavičiūtė et al., 2017). A study by Lou demonstrated that TNTs could facilitate the intercellular spread of therapeutic oncolytic viral vectors; furthermore, TNTs also mediated the bystander effect by facilitating distribution of therapeutic drugs (nucleoside analogs) activated by viral thymidine kinase. The study establishes TNTs as an alternate route, beyond gap junctions, for cells to amplify the effects of potential disease-targeting drugs, opening a new door to harnessing TNTs as potential cellular conduits for drug delivery (Bruzauskaite et al., 2016; Antanavičiūtė et al., 2017). Conversely, where infectious agents ‘hijack’ TNTs to spread their pathology, blocking TNTs by targeting specific TNT components could represent another therapeutic strategy in disease. Thus, further research in this area is required to help the scientific field to understand this dual nature of TNTs better.

## Conclusions: prospects for TNT biology, gap junctions, and translational research

There is a growing body of evidence that supports the critical role of TNT-like structures and gap junctions in development, immune response, and disease. The increased TNT formation in several pathogenic conditions provides a unique opportunity to pharmacologically modulate these processes to block or increase their formation to control the spread of pathogenic and healthy components communicated through TNTs.

An overview of recent scientific literature indicates that TNT-gap junctional research is in its early stage of research and there are still a number of outstanding questions relating to the mechanisms and signals driving the formation of TNTs, their morphology and detailed structural organization, their components (e.g., proteins and lipids), mechanisms determining their permeability and cargo, how TNTs collapse, biomarkers of TNT formation, and, most importantly, how all of these factors are associated with particular cellular functions (Figure 1). However, it is clear that the main function of TNTs during adulthood is to participate in the immune response and during several pathological conditions. To address these key TNT-related questions, a collaboration between leading TNT scientists is vital, and several aspects and questions of this emerging field are summarized in Table 1. Also, GJ not only communicate to neighboring cells but also potentially through TNTs over a long-range.

**Table 1:** Open questions in the area of TNT and gap junctions.

**Theme**	**Specific questions**	**Workshop output**
Pathophysiological function of TNTs	Why are TNTs induced in disease?	Hijacking development and/or an evolutionary response Stress induction Specificity in cargo delivery—energy conservation Exchange of genetic material to support disease or rescue damage cells from cell death
Translational relevance of TNTs in disease	TNTs are thought to play a role in disease—which disease(s)?	Diseases include cancer, neurodegenerative disorders, tau related diseases, HIV, lysosomal disorders, inflammation, parasitic infections (Malaria)
	What role do TNTs play is disease?	Promoting the disease (e.g., spread of virus, protein aggregates), mitochondria between cancer cells (chemo-resistance) Rescuing the cell function (e.g., lysosomal and mitochondrial transfer to defective cells)
	Elucidating Normal Physiologic functions of TNTs	Facilitating cell contact during development (e.g., cytonemes) Promoting cell communication (e.g., Signaling) between distant cells Immune response and organelle exchange mechanisms Function in stem cell biology and tissue repair Diverse heterogeneity of TNTs (phenotype/functions/disease/health) Proven importance/roles of TNTs for immune response Multi-functional cargo (“FedEx”-like)
	What are the key learnings	Cell structure is important for spread/progression of the disease by transferring, e.g., infectious agents between cells and for cell-to-cell communication, e.g., during development, tissue regeneration Potential therapeutic target to block disease progression TNT formation during development (e.g., CNS), pathological events (pathogens, tumor cells, misfolded/aggregated/stress protein), during regeneration process (stroke), in inflammation/immune response and drug delivery Importance of identifying mechanism of actin/motors that drive TNTs Elevating research beyond *in vitro, in vivo* and 3D studies Importance of examining heterotypic TNT interactions, e.g., cancer-to-stem cells, cancer/stroma To examine the immense heterogeneity of definition of TNTs
Cellular mechanisms of TNTs	What is known about TNT cell biology?	Strong evidence of TNT formation *in vitro* (e.g., infectious disease, oncology, neurology, development) Intercellular communication /signaling/cargo/dyes Inducible (infection/inflammation) A Large variety of cells capable of TNT formation Some evidence of TNT formation *in vivo* (oncology) Evidence that M-sec, myosins, F-actin, and calcium transfer are involved Shaking/physical disruption blocks TNT formation Gap junctions may play a role in TNT connecting to receiving cells (Focus if this review)
	What is the overlap, and what are the potential differences, of TNT biology in normal cells vs. in disease, and between different diseases?	Differences—induction of TNT seems to be associated with “diseased” cells The direction of cargo communication Cell types/microenvironment (tumor/inflammation) In diseased cells, F-actin polymerization is increased.
	How does a donor cell “decide” what organelles, molecules or signals transfer through TNTs?	Key factors include: ◦ Variety of TNTs ◦ Different triggering factors (pathogen, metabolic stress, e.g., reactive oxygen species) ◦ Selectivity of organelles and direction of travel ◦ Uni-/bi-directional depends on cell type &/or cargo Stress response Preferential transfer of mitochondria Virus hijack TNTs
	TNT research has advanced over the last 10 years—what are the key focus areas to advance this science?	Mechanism of TNT formation—trigger, direction, cargo/content, structure, response Better characterization—different types of TNTs, types of cells able to make TNTs *In vivo* evidence of TNT—in development, in disease model, regeneration mechanism (stem cells) Develop/test TNT blockade strategies and TNT induction mechanism Delineate relationship between TNTs and inflammation/immune response stromal The following items are needed: ◦ Chemical tools ◦ Common mechanism of transfer ◦ Selectivity ◦ A TNT biomarker ◦ *In vivo* evidence Cargo identification Regulation and induction/suppression Accept TNT heterogeneity (no simple narrow definition to make this science grow) Technology hurdles: Need higher resolution microscopy (e.g., EM, cryoEM, organelle level resolution, identifying cellular structure “signatures”) Collaboration with medicinal chemists to synthesize inhibitors of key TNT-drivers (e.g., M-sec) Proteomics to identify TNTs and their contents Targeted drug delivery via TNTs (e.g., siRNA) “How does the TNT know where to go?” cell sensing mechanisms? Translational relevance: identify strategic approaches that are disease specific Standardization of terminology Broader definition, including subtype descriptors *In vivo* data, especially patient data Better, specific markers → enable 3D culture experiments, *in vivo*, etc TNT biochemistry—reconstitute in a cell-free system

Thus, by blocking TNTs and/or gap junctional communication at long distances in infected cells and disrupting the transmission of infectious material to neighboring cells, this approach represents a unique therapeutic strategy for some hard-to-treat diseases which includes some retroviral and microbial infections, neurodegenerative disorders and metastasis in certain cancers.

## Author contributions

All authors listed have made a substantial, direct and intellectual contribution to the work, and approved it for publication.

### Conflict of interest statement

The authors declare that the research was conducted in the absence of any commercial or financial relationships that could be construed as a potential conflict of interest.
